# Governance systems for skilled health worker migration, their public value and competing priorities: an interpretive scoping review

**DOI:** 10.1080/16549716.2021.2013600

**Published:** 2022-01-17

**Authors:** Kenneth Yakubu, Andrea Durbach, Alexandra van Waes, Sikhumbuzo A. Mabunda, David Peiris, Janani Shanthosh, Rohina Joshi

**Affiliations:** a Faculty of Medicine, The George Institute for Global Health, Faculty of Medicine, University of New South Wales, Sydney, Australia; b Australian Human Rights Institute, Faculty of Law and Justice, University of New South Wales, Sydney, Australia; c Department of Health Sciences, Boston University, Boston, MA, USA; d School of Population Health, Faculty of Medicine, University of New South Wales, Sydney, Australia; e Health Systems Science, The George Institute for Global Health India, New Delhi, India

**Keywords:** Health workforce, governance, emigration, Immigration

## Abstract

**Background:**

Governments worldwide participate in skilled health worker (SHW) migration agreements to protect access to health services in their countries. Previous studies have described the value offered by these agreements in separate source and destination country perspectives.

**Objective:**

We sought to identify and summarise existing literature on shared value creation for both source and destination countries.

**Method:**

We conducted a systematic scoping review of health databases and grey literature. Using Mark Moore and Colin Talbot frameworks, we summarised the public value propositions in migration agreements and how state actors addressed competing priorities.

**Results:**

Most articles (34/63, 53.9%) reported transnational SHW mobility as the public value proposition for source and destination countries. Fewer articles, 14 (22.2%) and 9 (14.3%) articles, respectively, referred to achieving health workforce sustainability and building capacity for SHW migration governance as shared public values. The most identified competing public value (CPV) was an overriding focus on addressing SHW shortages in destination countries (20/63, 37.7%). Efforts to address this CPV include mitigation of the adverse effects of migration (43/63, 68.3%) and promoting health workforce development in source countries (15, 23.8%). At the same time, state actors retained regulatory discretion for protecting their country’s public health interest (34, 54%). Most articles reported the lack of funds (15/63, 23.8%) and implementation mechanisms (19/63, 30.2%) as constraints on the authorising environment and the operational capacity of SHW migration governance systems.

**Conclusion:**

Regarding SHW migration governance, the literature reports shared public value propositions for source and destination countries. It also shows how the value-creating process in the management of SHW migration favours destination countries. Future studies will need to explore shared value creation models that ensure equity in the governance of SHW migration.

## Background

Central to a health system’s ability to offer value is the number of skilled health workers (SHWs) relative to the patient population, their competence, and motivation to provide quality health services [1,2]. Globally, there is a shortage of SHWs, and the acuteness of this problem differs between health systems in low, middle-income, and high-income countries [3]. To cope with the shortage of SHWs, many high-income countries (HICs) have relied on the immigration of foreign-trained SHWs. In 2000, the total number of foreign-born doctors who migrated to countries belonging to the Organization for Economic Development (OECD) was 415,936. By 2015 this had risen to 716,432, reflecting a migration growth rate of around 50% [4]. Between 2009 and 2016, foreign-trained nurses in Australia increased from 14% to 18.2%, and in New Zealand, from 14.7% in 2002 to 26.7% in 2016. For the Gulf states, foreign-trained nurses increased to as much as 79% in 2008 [5]. The recruitment policies of governments in high-income countries (HICs) have worsened health workforce shortages in LMICs and failed to promote collective efforts towards addressing a global problem [3,4].

By building on previous SHW migration initiatives (e.g. the UK Code [6], and the Commonwealth Code on the International Recruitment of Health Workers [7]), the World Health Organization (WHO) has attempted to address health workforce inequities through its Global Code of Practice on the International Recruitment of Health Workers (WHO Code) [8]. In 2010, the World Health Assembly adopted the WHO Code as a consensus approach for handling international recruitment of SHWs while remaining sensitive to the needs of source countries and the migratory rights of individual health professionals. The Code promotes transparency, fairness, mutuality of benefits, compensation, and reparation in the migration of SHWs. Although directed at addressing the global challenges of health worker migration, the Code has achieved varying degrees of compliance – primarily due to its non-binding nature and competing interests between member states [9].

To ensure shared value creation for all countries involved in current SHW migration governance systems [10], an assessment of previous migration initiatives is necessary. The public value theory by Mark Moore is helpful in this regard. It defines public value as the desired social outcomes generated by governments through services, laws, and regulations [11,12]. Moore recommends that state actors communicate a clear value proposition (i.e. how their actions will improve a public problem), build legitimacy and support (so they can secure a flow of resources), and acquire relevant operational capacity [11,12]. Since global governance systems for SHW migration include bilateral, regional, and international initiatives, assessing how these systems create public value requires a review of how state actors navigate a shared value-creating process, as well as how they handle competing interests.

### Aim and objectives

This review aimed to identify and summarise available literature on the public value offered by global governance systems for SHW migration. We focused on describing (1) the public value propositions by various global governance systems for SHW migration, (2) how state actors handled competing public value propositions, and (3) what enabled and constrained the achievement of these propositions.

## Methods

We registered the protocol for this scoping review on the Open Science Framework platform [13] and reported our findings according to the Preferred Reporting Items for Systematic Reviews and Meta-analyses (PRISMA) [14]. Our population of interest was SHWs worldwide. To capture the concept, we focused on public value propositions (i.e. how various sponsors of a migration policy/program communicated what was essential to both source and destination countries). For context, we considered multiple governance systems for SHW migration (e.g. bilateral, regional, and international governance systems). We used a qualitative content analysis approach to synthesise our findings since this is more suitable for mapping existing literature and defining knowledge gaps [15].

Mark Moore’s Strategic Triangle Framework [12,16] and Talbot’s Competing Public Values model [17] guided our engagement with the literature and classification of the broad content areas. Moore recommends that governments provide a clear value proposition for the problem they wish to solve. He also advised that they build legitimacy and support (by ensuring that relevant stakeholders agree with their value propositions and can guarantee a flow of resources for their realisation) and acquire relevant operational capacity (the know-how and ability to achieve the desired outcome) [12]. Previous studies have used the public value theory for analysing engagements between governments and their citizens. Hence, the public sphere is often nationally defined, and its guiding principle is democratic governance [18]. However, for this review, we expanded the definition of the public sphere to mean a ‘global public sphere.’ We focused on how various state parties navigated a shared value-creating process in SHW migration governance (referring to what adds value to a source-destination country dyad).

There are often competing public value propositions at a global forum where state parties discuss what delivers value to their countries. Talbot’s Competing Public Values Framework [17] describes how state parties can choose to resolve this conflict by either focusing on a shared sense of identity or cooperating on issues of common interest (solidarity). State parties can also choose to pursue their interests, thus competing for scarce resources (autonomy), subscribe to a collective understanding of ethical standards (equality and equity), or submit to a regulatory mechanism perceived to be reliable, efficient, and fair (authority) [17].

### Eligibility

To be included in the review, we considered articles that mentioned migration among doctors, pharmacists, nurses, and dentists (i.e. health professional groups that are often mentioned in the migration literature). We also included articles that discussed the design or implementation of SHW migration agreements (policies, regulation, management, planning, or governance decisions) between two or more countries. We excluded articles that only described SHW migration policy engagements within one country or focused only on the drivers of SHW migration. We also excluded those that described the consequences of SHW migration or concentrated only on policy recommendations without mentioning actual implemented policies or initiatives.

### Information sources and search

With the help of a Librarian at the University of New South Wales, we identified relevant keywords and tested our search strategy in the Global Health database. We adapted the search terms for the following databases: Embase, CINAHL, Ovid MEDLINE(R), EMcare and Epub Ahead of Print, In-Process & Other Non-Indexed Citations and Daily. We also checked the reference list of included studies and adapted the search strategy for Google Scholar and the World Health Organisation International Repository for Information Sharing (WHO|IRIS). Most SHW migration-related policies were written and implemented after the mid-90s [19]; hence, we limited our search range to articles published from 1990 till 2020.

We used Zotero Online for data management and ensured that the authors had access to the same articles. The complete electronic search strategy is available in the supplementary file (Appendix 1).

### Selection of sources of evidence

The lead author (KY) removed duplicates from the initial yield of articles and created a list of the remaining papers. The review authors (KY and AVW) independently screened the titles to remove those unrelated to the study’s aim. We independently screened abstracts from the remaining articles and included those that mentioned a SHW migration initiative where state parties from two or more countries made collective decisions. When the relevance of a paper was not clear, we read the full text before deciding. We discussed discrepancies at each stage of the screening process, and whenever there was a failure to achieve consensus between KY and AVW, the third author (SAM) intervened.

### Data charting process and data items

The lead author recorded information for each article, such as name of authors, title, year of publication, and the study design on a spreadsheet. He also created a codebook based on Moore’s Strategic Triangle Framework [12,16] and Talbot’s Competing Public Values model [17]. The codebook described how to identify public value propositions, competing public values, how state parties addressed these, as well as enabling and constraining factors. The three authors (KY, SAM and AVW) discussed the codebook and agreed on a final copy. After that, they independently and manually annotated portions of each article relevant to the study objectives using the codebook and a deductive approach. They also employed an open coding approach to capture relevant parts of the data that fit under the broad categories described by the two frameworks. After discussing and resolving discrepancies at this stage, KY imported the annotated articles into the NVivo© software and applied the agreed-upon codes. We have provided details of the codebook in Appendix 3.

### Synthesis of results

The lead author synthesised the data by placing similar codes into categories and provided a narrative review of information relevant to the study objectives. In line with existing recommendations on scoping reviews, we did not conduct a critical appraisal of individual studies since this review aimed to summarise existing literature and identify knowledge gaps [14,20].

## Results

### Selection and characteristics of sources of evidence

**Figure 1.**
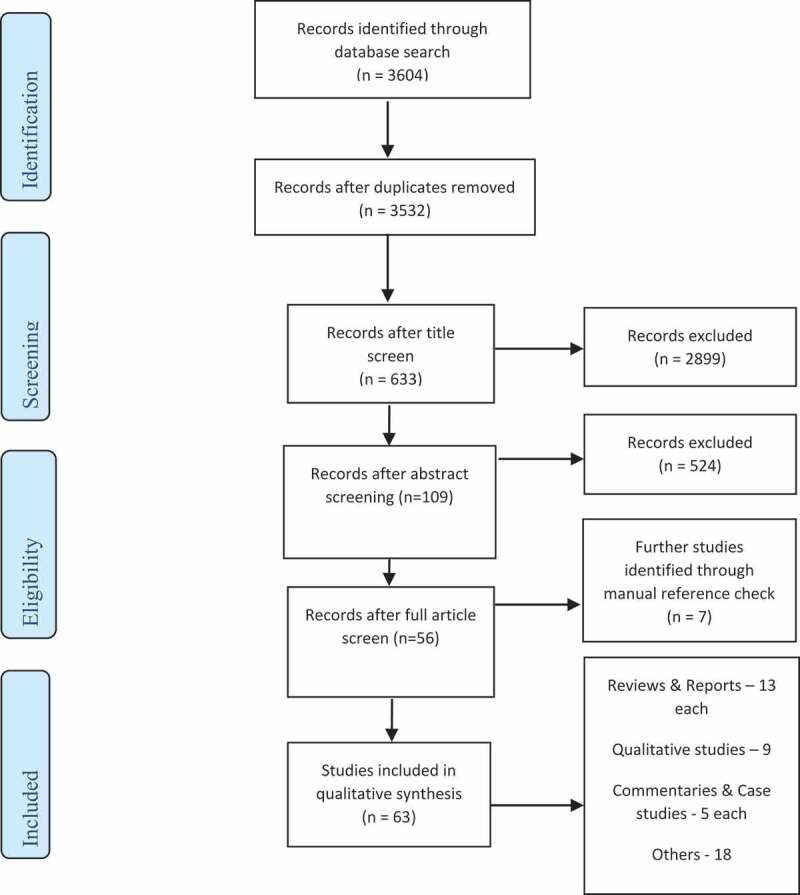
Summary of search, selection and inclusion process.

**Figure 2.**
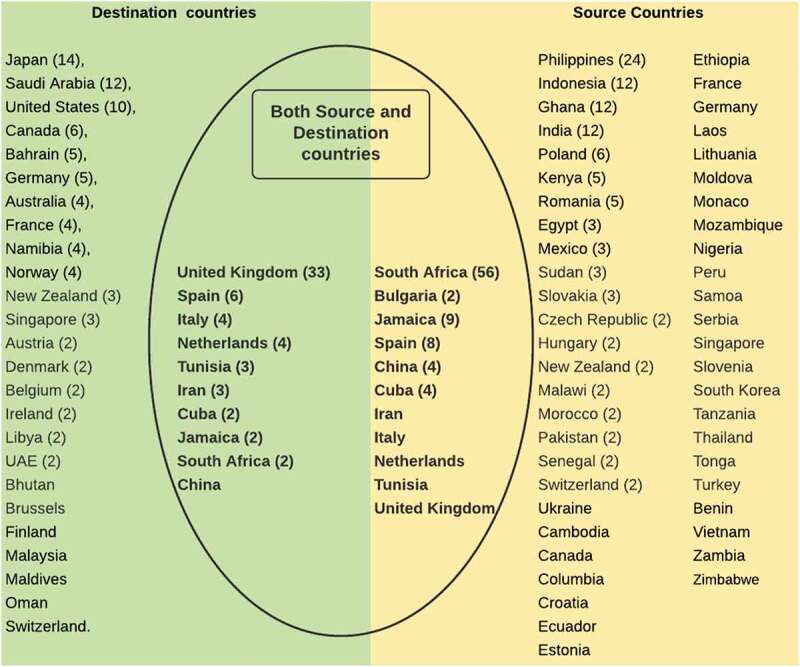
List of countries reflected in the reviews.

We retrieved a total of 3604 articles, out of which 63 were finally included in the review (see Figure 1). These studies were published between 1993 and 2020. Most articles were reviews or reports (13/63, 20.6%), mentioning South Africa and the UK more frequently than other countries (see Figure 2). While other studies have used the term ‘Brain Drain’, we consider this contentious and dated and refer instead to SHW migration in this paper. We have summarised content areas relevant to our objectives and provided details of included articles in Table 1 and Appendix 2, respectively.

**Table 1.** Distribution of content areas from the primary studies included in the review

Area of inquiry based on the framework	Category	Sub-categories	Number of articles (%)
Public Value proposition			
	Facilitate SHW mobility between countries	-Part of broad initiatives aimed at securing economic value for both source & destination countries -Addressing SHW unemployment in source countries, and workforce shortages in destination countries. -Maintaining health services near shared national borders. -Comparability of competences, and integration of health systems.	34 (53.9%)
	Achieve health workforce sustainability	-Track workforce needs and migration flows. -Health workforce development, and their distribution -Coordinate labour market activities	14 (22.2%)
	Build capacity for SHW migration governance	-Monitoring of migration flows and their impact -Identification & dissemination of best practices -Mechanisms for monitoring implementation	9 (14.3%)
Competing Public Values			
	Patient safety in destination countries	-Concerns about quality of SHWs attracted, and its impact on patient safety	10 (15.9%)
	SHW shortages in destination countries	-Addressing SHW shortages in destination countries, less concern on impact in source countries	15 (23.8%)
Handling Competing Public Values			
	Equality and equity	-Mitigating negative effects of migration -Health workforce development	43 (68.3%) 15 (23.8%)
	Protecting a country’s autonomy	-State parties exercise regulatory discretion to protect their county’s public health interests.	34 (54.0%)
	A focus on cooperation	-Seeking to forge solidarity between state parties	18 (28.6%)
	Delegating authority to a regional governance system	-A regional council is recognised as the unbiased arbiter on competing interests between state parties	3 (4.8%)
Enabling/Constraining factors			
	Authorising environment	Alignment of interests/prevailing social pressure Funds Data Technical resources	4 (6.3%) 15 (23.8%) 12 (19.0%) 3 (4.8%)
	Capacity to harness available resources	Implementation mechanisms Monitoring ability	19 (30.2%) 4 (6.3%)

*SHW – Skilled Health Workers

### Public value propositions

**Figure 3.**
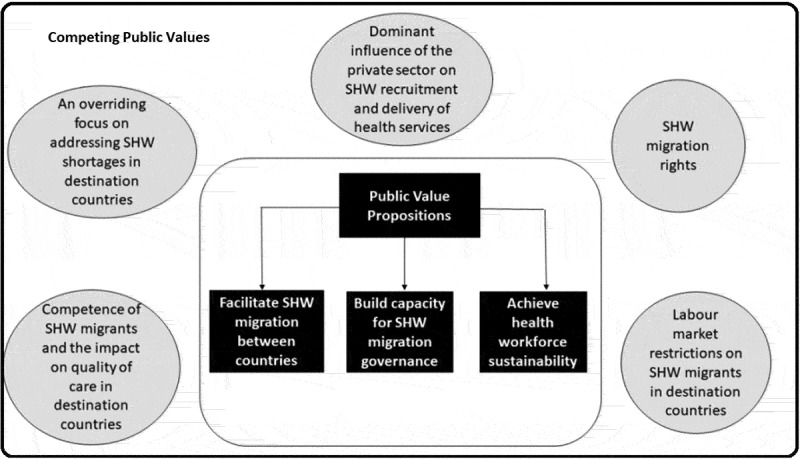
Public values and competing public values in SHW migration governance.

We reviewed 55 bilateral, 33 regional, and 13 international migration policies/initiatives and identified three content areas that describe the public value propositions of SHW migration governance programs/initiatives in the reviewed literature (Figure 3). We provide a further description of these content areas below:

#### Facilitating SHW mobility between countries

The removal of barriers to SHW mobility was a public value proposition described by most of the studies (34/63, 53.9%) in this review. Twenty-two (34.9%) articles captured this as part of regional migration initiatives [21–42], 15 (23.8%) within bilateral [22,24,25,30,32,34,36,37,41,43–48] and 14 (22.2%) within international initiatives [22,24,31,33–36,43,49–54]. The initiatives include managed migration programs aimed at addressing SHW unemployment in source countries and health workforce shortages in destination countries (e.g. between Kenya and Namibia; the UK and South Africa) [22,32,35,37,38,43,44,46,52,55–60]. Formal migration agreements also occurred between countries at similar income levels, where bilateral [22,30,41], and regional agreements [29–31,37–41] were necessary for mutual recognition of professional qualifications, and integration of different health systems.

The competing public values include concerns about the quality of SHWs attracted by these formal agreements and their impact on patient safety in the destination countries (10/63, 15.9%) [23,24,32,33,37,40,41,46,47,61]. Another competing interest for destination countries was the need to protect their workforce despite existing agreements aimed at facilitating the migration of SHWs. Focusing exclusively on the interests of a domestic workforce affected the labour rights of migrating SHWs, i.e. recognition of their qualifications, fair remuneration and opportunities for career progression [24,26,29,31,32,46,47]. There were also concerns about the rising dominance of private organisations on the recruitment of SHWs, and the delivery of health services [62].

When the preference of destination countries was circular or temporary migration, this was a competing priority for many SHWs in source countries who wanted permanent migration [45,48]. Also, many bilateral [22,32,34,36,37,44–46,48], regional [23,31,36,41,53] and international [31,36,43,53,54] agreements were influenced by the need to address SHW shortages in destination countries, and did not capture the impact of SHW mobility on health systems in source countries. Since social networks and family connections largely drive SHW migration, some studies described formal agreements as lacking the ability to equitably foster SHW mobility between source and destination countries [37] or lead to significant economic development in source countries [28,63,64].

#### Achieving health workforce sustainability

A small proportion of the reviewed articles (14/63, 22.2%) reported multilateral discussions on health workforce sustainability. Ten (15.9%) articles captured this as part of international migration initiatives [22,27,31,36,37,59,60,64–66], and 4 (6.3%) within regional initiatives [22,34,36,44]. These regional and international initiatives encouraged state parties to meet their health workforce needs from resources available in their countries. Most of the articles (10/14) acknowledged that this requires proper documentation of each country’s workforce needs and migration flows [27,31,34,36,37,44,60,64,66]. The need to improve health workforce development, coordinate health labour market activities, and address maldistribution of SHWs were also identified as critical priorities for ensuring health workforce sustainability [22,59]. Importantly, even though these articles clearly outlined a priority plan, none mentioned the actual implementation of these priorities among participating countries.

#### Build capacity for SHW migration governance

A small collection of the articles (9/63, 14.3%) described international migration initiatives to improve the capacity of current SHW migration governance systems. These initiatives included effective monitoring of migration flows and their impact, identification, and dissemination of best practices in SHW migration governance, as well as mechanisms for monitoring their implementation [9,31,32,36,48,58,62,64,67]. Out of the nine studies focused on this area, only one mentioned that formal agreements between state parties enabled a better understanding of health worker shortages and the multifaceted governance approaches required [36].

### Handling competing public values

**Figure 4.**
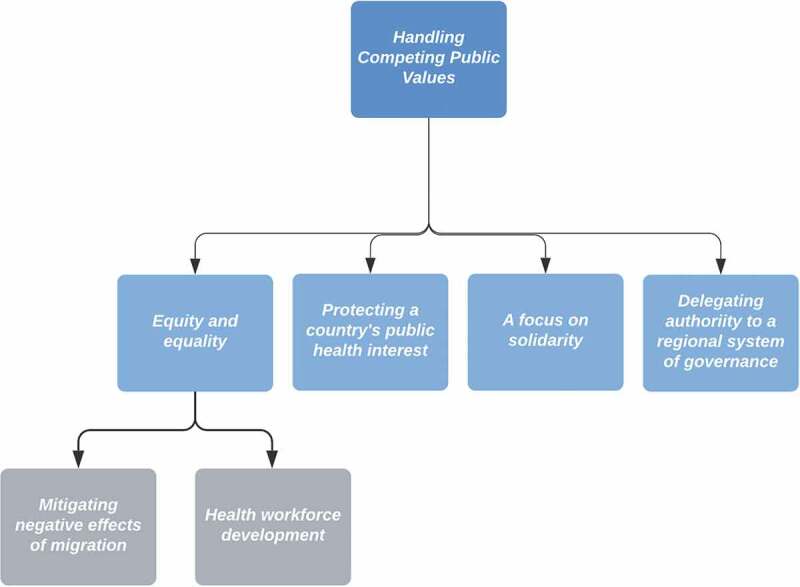
Handling competing public values in the global governance of SHW migration.

Four content areas (with their sub-categories) capture how state parties addressed competing public values in this area (Figure 4). These mainly occurred around facilitating SHW mobility between countries.

#### Equity and equality

Most of the articles (47/63, 74.6%) observed that state parties could address competing priorities in SHW migration governance by adhering to principles of equity and equality. They focused on the following two sub-categories:

##### Mitigating adverse effects of migration

Informed by concerns about the adverse effects of SHW migration in source countries, bilateral [22,24,31,34,36,41,42,44–46,48,55,56,58,68–71], regional [27,31,32,34,35,37,44,55,57,67,72] and mostly international [22,24,26,31,32,35–37,39,41,46,48,50–52,55,59,62,63,66,68,73–75] agreements discouraged active recruitment from countries with critical SHW shortages. Circular/temporary migration has been promoted as a way of providing a temporary solution to the issues of SHW unemployment, skills, and technology deficit, as well as promoting trade and economic development in source countries. It is not clear though, the extent to which this has led to health system strengthening or national development in source countries [31,32,36,37,46,48,53,61].

There were also proposals to limit the number of SHWs that could be recruited and provide compensation to source countries for the loss of their SHWs. These migration agreements also captured the need to ensure fair treatment of SHWs when they migrate and ensure the return of skills, experiences, and development back to source countries [22,24,27,31,34,35,37,41,44–46,48,50–52,55,57–59,63,66,72,74,75].

Although such agreements are laudable in intent, a significant number of articles (19/47, 40.4%) reported state parties’ failure to fully implement them. To the extent that these agreements were implemented, they did little to improve the functioning of health systems in source countries [22,24,29,31,36,37,46,48,52,53,61,64,68–70,72,75–77]. In addition, globalisation of the labour market [31,36,52] has distracted state actors in destination countries from promoting the right to health in source countries [32,35,36,41,55,70]. It has also prevented achieving consensus on the provision of compensation to source countries for the loss of their SHWs [31,35,36,44,48,50,55,65,69,73,78].

##### Health workforce development

Other examples showcasing the application of the principle of equity and equality include bilateral [9,37,44,79] and international initiatives [9,24,36] in which destination countries supported health workforce development in source countries. These migration initiatives include providing financial and technical support for establishing health professions where they do not yet exist, health staff exchanges, and improving medical curricula to meet national health needs in source countries [9,22,24,31,36,37,44,79].

Out of the 15 articles describing these health workforce development efforts, four (4/15) reported positive outcomes. These include migration initiatives that enabled an exchange of skills and knowledge between SHWs in source and destination countries, leading to improved quality of care in their respective countries [23,25,53,55]. It also includes initiatives that increased training opportunities for SHWs in source countries with limited training capacity [23]. For SHWs who migrated to receive further training, some eventually returned to their countries with new skills, increasing their income in the process [32,53,55]. However, four studies (4/15) found that return migration was not always achievable, as workforce development was either driven by individual SHW effort [34] or by migration policies that did not deter SHWs from remaining in the destination countries [53,55,79]. Even when SHWs returned to their home countries, one article suggested that the experience gained may have been of little value due to the differences in contexts of practice [68]. One article stated that bilateral agreements on migration increased training opportunities for SHWs in a source country, but this was primarily private-sector driven [24]. With poor regulatory control over the quality of training offered at these private institutions, there was an over-supply of SHWs in the source country, and concerns about the quality of SHWs supplied to domestic and international labour markets [24].

#### Protecting a country’s autonomy

Participation in multilateral agreements did not stop state parties from retaining regulatory discretion over how migration policies affected SHWs availability in their countries [23,24,26,29–33,36,39,40,45,50,51,74,80,81]. Even when state parties chose to prioritise what was in the best interests of their countries, it was not always clear whether this was valued by their citizens [23,29,31,34,36,37,41,43,46,47,50,51,53–55,58,61,64,65,72,75,76].

#### Delegating authority

Referring to migration initiatives in Europe, a few articles (3/63, 4.8%) described how its regional system of governance (to which state parties had delegated their authority) was effective for addressing competing public values. The European Union (EU) promotes rules and regulations that bind its 27 member countries to economic cooperation and development [23,32,40]. The EU system allows member states to debate economic policies (including skilled health migration policies) at its Parliament and Council. The European Commission makes the final decision and requires member states to incorporate it into their domestic laws within a specific period [40].

#### A focus on cooperation

Competing public value propositions were also resolved by identifying areas of cooperation and making decisions that protected cohesion within a group of participating countries (18/63, 28.6%). This approach characterised regional or international initiatives where member states had a common sense of identity or purpose [23,26,27,32,34–36,38,48,58,61,62]. Where these were absent (i.e. a common sense of identity or purpose), it was difficult to align varying perceptions surrounding SHW migration governance, thereby limiting the legitimacy and support for any agreement that was signed [9,35,36,43,55,64,67,69].

### Enabling and constraining factors

Achieving public value and addressing competing priorities within these formal SHW migration agreements were enabled or constrained by the adequacy of resources provided by participating source and destination countries and their ability to harness them.

#### Adequacy of resources

Half of the articles (7/15, 46.7%) that described adequacy of funds also reported that participating source and destination countries could fund their respective roles and responsibilities in formal migration agreements [27,32,34–37,82]. However, a majority (11/15, 73.3%) mentioned the absence of funds as a constraint [28,35,36,38,44,46,47,53,64,67,82]. For source countries, the lack of funds contributed to their reliance on destination countries for human resource development, migration and health systems strengthening [44,53,64]. For destination countries, more funding was needed to strengthen systems for foreign SHW recruitment. The constant demand for funds raised concerns about the sustainability of formal recruiting agreements [38,46].

Three studies (4.8%) stated that international organisations provided technical assistance to state actors on managing SHW migration [31,64,74] but struggled with low human resources to do this sustainably [36]. Most of the studies that mentioned data availability for achieving a stated public value proposition (9/12) also mentioned a lack of investment in relevant data systems by source and destination countries. This lack of investment in data systems hindered implementation of policies aimed at supporting SHW migration governance [9,24,25,29,35,36,53,55,68]. Where data was available, it was difficult to compare or share them with relevant stakeholders, making governance mechanisms between source and destination countries difficult [29,40,48].

#### Capacity to harness available resources

Eighteen articles (18/63, 28.6%) provided information on the implementation mechanisms of governance initiatives/programs for SHW migration. Eight of these (8/18, 44.4%) mentioned that the governance initiatives/programs depended on the voluntary ratification of participating state parties [25,31,44,48,60,63,66,78]. There were concerns that both source and destination countries had limited capacity for monitoring the implementation of agreements, especially their limited ability to capture the activities of private recruiters [33,36,48,55].

A lack of relevant domestic legislative frameworks, absence of a shared understanding of factors related to SHW migration [29,55,67], language barriers, different qualification/competency systems, and poor migrant support further characterised the implementation mechanisms of multinational migration agreements [24,25,30,33,41,46–48,59,62,78]. Differences in medical training and practice between countries hindered mutual benefits from SHW migration [9,31,36,38,46]. One study mentioned that the responsibility for monitoring these agreements fell on the destination countries, who were often unable to monitor the impact of these agreements on source countries or were not motivated to do so [55].

Implementation of formal agreements on SHW migration achieved positive results when interests between source and destination countries aligned and when state actors sought to avoid high political costs associated with competing priorities (4/18, 22.2%). An example of the first instance (i.e. alignment of interests between source and destination countries) is reflected in the negotiations between the Philippines (a source country that sought to make economic gains from migration) and the UK (a destination country that sought to meet their workforce deficits) [58]. In addition, source and destination countries were more likely to facilitate SHW mobility between their countries if they had a long history of working and learning from each other and had taken time to build the necessary infrastructure for implementing recruitment agreements [32,36,58].

In the second instance (i.e. avoiding high political costs associated with competing priorities), when there were strong international and domestic social movements that condemned the action of one state party (e.g. international and domestic outcry against UK’s recruitment behaviour), the political pressure this generated influenced state actors’ actions towards principles of equity and equality [32,36,58]. Similarly, it was easy to promote principles of equity and equality when there were concerns about diseases that posed public health threats to both source and destination countries [53].

## Discussion

We set out to provide a review of shared value creation and the management of competing priorities in the global governance of SHW migration. This review revealed that the value-creating process of SHW migration governance systems often prioritised the workforce needs of destination countries (largely HICs).

Increasing SHW mobility for mutual benefit, achieving health workforce sustainability, and building capacity for SHW migration governance were the shared public value propositions applicable to source and destination countries. Except for an increase in some state actors’ understanding of the complexities underpinning workforce sustainability, none of the articles reported complete fulfilment of these public value propositions. In addition, we found that concerns for patient safety, quality of care and SHW shortages in destination countries, were considered at the expense of the right to health in source countries.

State parties addressed these competing values by mitigating the harmful effects of migration, supporting health workforce development in source countries, promoting cooperation between state parties, and delegating authority to a regional system of governance while exercising regulatory discretion to protect their country’s public health interests. Implementation mechanisms for these agreements and an authorising environment were significant factors influencing the attainment of the stated public value propositions and how state parties addressed competing priorities.

Looking at the pattern of public value propositions captured in this review, a substantial portion of the literature focused on short-to-medium term goals (i.e. economic gains, employment for a skilled health workforce) versus long term goals (a sustainable workforce and capacity for skilled health migration governance). This behavioural pattern (in choosing short-to-medium term public value options over long-term ones) might reflect the inter-temporal character of state actors represented in this review. State actors tend to choose short term over long term policy options when faced with either electoral insecurity, poor information on the social returns from a long-term policy, or a lack of institutional capacity for structuring costs and benefits of a policy decision [83,84].

It is also important to ask, ‘for whom was value created?’ It appears that concerning health workforce sustainability and migration governance capacity, this review shows that destination and source countries have not enjoyed much value. Regarding the need to facilitate SHW mobility, value creation occurred for many destination countries and SHWs migrating from source countries. This pattern of value creation exposes equity gaps within governance systems for skilled migration [85,86].

**Figures 5.**
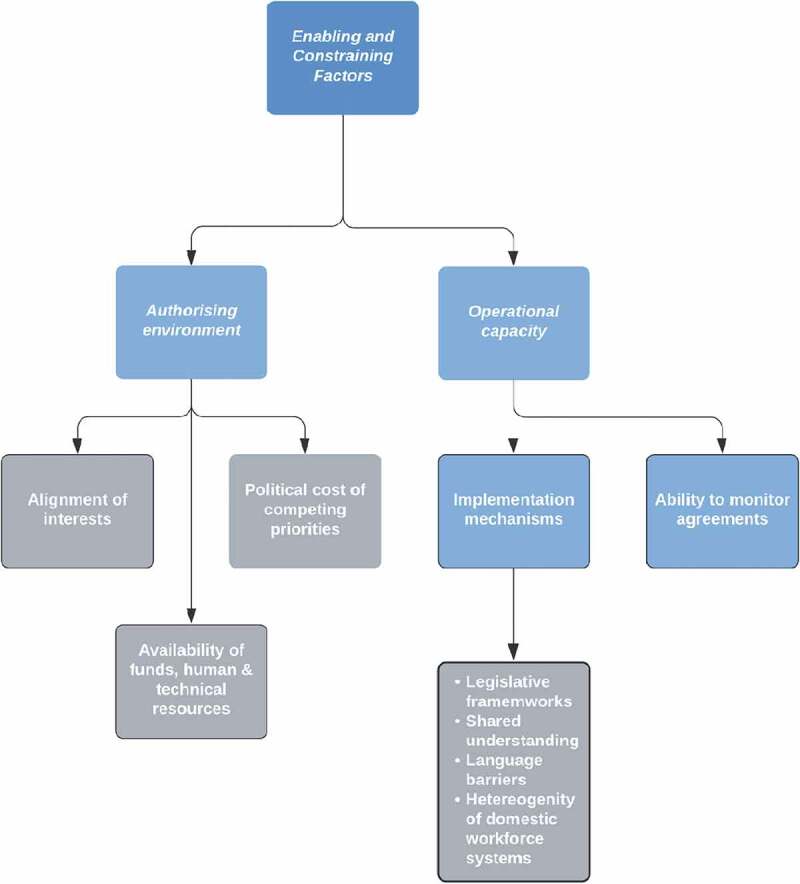
Enablers and constraints in creating public value and handling competing public values in the global governance of SHW migration.

Few studies offered information necessary for describing factors that impact a shared value-creating process in SHW migration governance. Where this information was available, a closer look at the constraints (funds, human and technical resources, voluntary nature of agreements, implementation mechanisms, and the ability to monitor agreements) and enablers (alignment of interests and the political cost of competing priorities) show that they map on to two domains of Moore’s Strategic Triangle framework (i.e. building legitimacy/support and operational capacity, see Figure 5).

Helgason (2014) adopts a similar approach for determining how healthy multilateral systems of cooperation can be achieved [87]. Even though Helgason (2014) considered all parts of Moore’s framework relevant, they focused more on authorising environments (achieved by building legitimacy and support). Helgason (2014) argued that an authorising environment legitimises a public value proposition and guarantees access to resources. Similarly, they opined that the authorising environment enables operational capacity for a policy [87].

A challenge remains to achieve such an authorising environment, seeing that the primary methods for handling competing public values as captured in this review (i.e. equity/equality versus autonomy) seem to pitch commitment to a domestic over a global public sphere. Other studies have identified state identities (i.e. shared beliefs within a country about itself and how it differs from how they perceive other countries) as the underlying causal mechanisms for this tension between the domestic and global public spheres [88]. These identities are not fixed, and through repeated interaction within a small group of actors, an evolution of cooperation can occur [88,89]. Our review suggests that this evolutionary process has occurred to a degree within the regional governance system in Europe.

However, we acknowledge that the COVID-19 pandemic might impact this evolutionary process of cooperation. At the onset of the pandemic, governments worldwide shut their national borders to control the spread of the COVID-19 virus. The closure of borders led to a break in the global supply of SHWs to destination countries [90]. As the pandemic progressed, states found ways to ease the migration of SHWs to address workforce shortages in their countries [91–93]. As a result, the COVID-19 pandemic has heightened concerns on competing public values in the governance of SHW migration, i.e. the overriding focus on the supply of SHWs to destination countries, less commitment to issues of health workforce sustainability and improving capacity for SHW migration governance [92].

Another challenge relates to the principles of equity and equality and their use for addressing competing public values in SHW migration governance. While equality refers to differences between individuals/groups based on a standard index, this becomes an issue of equity when unjust systems cause this difference [94]. A governance system that promotes equality might require that all SHWs have access to any domestic labour market and all governments should have the same opportunity to recruit from a global pool of SHWs. On the other hand, equity will require that since many (HIC) destination countries have enough resources to train the health workers they need, their recruitment of SHWs should not affect the health workforce needs of source countries.

The WHO has recommended 4.45 doctors, nurses, and midwives per 1000 population as the minimum health workforce requirement for attaining the sustainable development goals (SDGs) [95]. Hence, efforts to mitigate the adverse effects of migration and support health workforce development can be evaluated based on how it impacts this minimum benchmark in source countries. Since this is a global index, each migration initiative would need to be guided by the source countries’ national/subnational workforce needs.

It will be necessary to explore how value-creating models for global, regional, and domestic public spheres can co-exist during and beyond the COVID-19 pandemic. Seeing that global governance systems for SHW migration are not limited to hierarchical systems of governments, future studies will also need to consider the impact of communities, non-governmental organisations, and other stakeholders on the public value creation process.

## Strengths and limitations

This study was limited to public value propositions by state actors. However, the derived public value propositions may not be a true reflection of what the public considers valuable. Even though we used a systematic approach in our literature review, we acknowledge that many facets of global SHW migration governance and how it creates public value have not been fully documented in the literature. Our search did not yield relevant articles written in other languages. Retrieving only articles written in English might have been related to our search strategy. However, we consider our engagement with the literature innovative, going beyond discourse on health workforce needs in source countries to exploring the literature on shared value creation for a source-destination country dyad. Using a qualitative content analysis approach for this review allowed for a manifest and latent explanation of the literature.

## Conclusion

With regards to SHW migration governance, this review considered a shared value-creating process for a global public sphere. This approach does not ignore the sovereignty of individual states in creating domestic value. Instead, it acknowledges that the value-creating process for domestic, regional, and global public spheres can co-exist. This review revealed a pattern of value creation in SHW migration governance that favours destination countries more than the population in source countries. State parties were guided by equity and equality principles as they attempted to improve existing value creation patterns. In addition, they exercised regulatory discretion to ensure public health benefits for their countries. The review showed state actors’ behavioural patterns, suggesting a preference for short-to-medium term public value options. Our findings also revealed that formal migration agreements involve a lot of bureaucracy and time to set up. Furthermore, the activities of non-state actors (including private recruiters, family links and social networks) account for higher SHW migration flows than formal migration agreements.

Future studies/efforts should seek to expand an understanding of authorising environments and design implementation mechanisms for a shared value-creating process in the governance of SHW migration. A shared value-creating process for SHW migration governance will require the design of incentives for skilled labour markets – incentives that promote recognition of the right to health in source countries and harnesses inputs from state and non-state actors (e.g. private recruiters, civil society organisations, community groups). Future studies will also need to consider regional governance systems that oversee health workforce agreements, mutual economic benefits for participating state parties, and protection of the right to health for all.

## Paper context

Researchers often describe skilled health worker migration governance using separate source and destination country perspectives. This review identified transnational mobility of skilled health workers as a shared priority for source and destination countries. It recommends that future studies explore more opportunities for shared value creation within governance systems for skilled health worker migration.

## Appendix 1 Search strategy

*This search strategy was used for the Global Health database, and adapted for Embase, Emcare, MEDLINE(R) and Epub Ahead of Print, In-Process & Other Non-Indexed Citations, Daily and Versions(R)*

**Table:**

Migration
1. brain drain.mp. or migration.sh. or emigration.sh. or immigration.sh
Health professionals
2. (skilled and (health* or medical) and (profession* or personnel or staff or worker* or manpower or workforce)).mp.
3. nurse*.mp.
4. physician*.mp.
5. doctor*.mp.
6. dentist*.mp.
7. (midwife* or midwiv*).mp.
8. pharmacist.mp.
9. 2 or 3 or 4 or 5 or 6 or 7 or 8
Governance
10. (governance or government).sh.
11. administration.sh.
12. decision making.sh.
14. regulation.sh. or regulation*.mp.
15. (health policy or regional policy).sh. or policy.mp. or policies.mp. or policy.sh. or government policy.sh. or policy respons*.mp. or intervention*.mp. or agreements.mp. or regulation.mp.
16. planning.mp. or planning.sh. or work planning.sh.
17. 10 or 11 or 12 or 13 or 14 or 15 or 16
Health professionals AND Migration AND Governance
18. 1 and 9 and 17

**Google Scholar and WHO | IRIS**

(‘brain drain’ or migration or immigration or emigration) AND (skilled and (Health or Medical) AND (professional or personnel or staff or worker or workforce or manpower) or nurses or midwife or midwives or pharmacist or doctors or physicians) AND (governance or policy or regulation or agreement or intervention or response)

**CINAHL**

**Table:**

Migration
S1 ‘brain drain’“
S2 migration
S3 (MH ‘Emigration and Immigration/LJ/EI’)
S4 S1 or S2 or S3
Health professionals
S5 health professional
S6 health personnel
S7 health staff
S8 health worker
S9 health manpower
S10 “health workforce “
S11 nurse
S12 doctor
S13 physician
S14 pharmacist
S15 dentist
S16 S5 OR S6 OR S7 OR S8 OR S9 OR S10 OR S11 OR S12 OR S13 OR S14 OR S15
Governance
S17 (MH ‘State Government’) OR (MH ‘Government’) OR ‘governance’
S18 administration
S19 ‘decision making’
S20 (MH ‘Professional Regulation’) OR (MH ‘Rules and Regulations’) OR (MH ‘Government Regulations’) OR ‘regulation’
S21 (MH ‘Public Policy’) OR (MH ‘Policy Making’) OR (MH ‘Health Policy’) OR ‘policy’
S22 ‘intervention’
S23 (MH ‘International Business’) OR (MH ‘Negotiation’) OR ‘agreements’
S24 (MH ‘Strategic Planning’) OR ‘planning’
S25 S17 OR S18 OR S19 OR S20 OR S21 OR S22 OR S22 OR S23 OR S24
Health professionals AND Migration AND Governance
S30 S4 AND S16 AND S25

## Appendix 2 Characteristics of each article and distribution of content areas

**Table:**

			Content areas (Bullet point (•) indicates a content area identified in each article)				
First Author (Year of Publication)	Study design /article type	Level of governance	PVP	AE	OC	CPV^¥^	HaCPV^¥^
Abuagla, A. (2016)(1)	Case study	Bilateral International	*	*	*	*	
Arnold, P.C. (2011)(2)	Essay	Bilateral	*		*	*	
Balasubramanian, M. (2016)(3)	Qualitative study	Bilateral Regional International	*	*	*		
Balasubramanian, M. (2011)(4)	Qualitative study	International					
Bidwell, P. (2014)(5)	Qualitative Content Analysis	Bilateral	*		*		
Brush, B.L. (2008)(6)	Integrative Literature Review	Bilateral International	*	*			
Brush, B.L. (2010)(7)	Historical analysis	Bilateral	*			*	
Buchan, J. (2010)(8)	Analytical Review	International			*	*	
Buchan, J. (2011)(9)	Editorial	International	*		*	*	
Buchan, J. (2003)(10)	Report	Regional, International	*	*			
Cheng, M.H. (2009)(11)	Report	Bilateral International	*			*	
Connell, J. (2011)(12)	Analytical Review	Bilateral Regional International	*	*	*	*	*
Cowan, D. (2006)(13)	Editorial	Regional	*	*	*		
Dhillon, I.S. (2010)(14)	Book	Bilateral Regional International	*	*	*	*	
Ennis, C.A. (2018)(15)	Transnational Analysis	Bilateral Regional, International	*	*		*	
European Observatory on HealthSystems and Policies (2014)(16)	Book	Bilateral Regional International	*	*	*		
Gerlinger, T. (2007)(17)	Analytical Review	Regional	*	*	*	*	*
Hawthorne, L. (2015)(18)	Project report	Bilateral Regional International	*	*	*	*	
Jourdain, A. (2017)(19)	Review	Regional International	*				*
Kalipeni, E. (2012)(20)	Analytical Review	Bilateral					
Kanchanachitra, C. (2011)(21)	Review	Bilateral Regional International	*	*	*		*
Kingma, M. (2001)(22)	Commentary	Bilateral Regional International	*	*		*	
Kingma, M. (2006)(23)	Meeting Presentation	Bilateral Regional International	*		*	*	
Kupfer, L. (2004)(24)	Program report	Bilateral Regional International	*	*		*	
Lewis, P. (2011)(25)	Case Study	Bilateral Regional International	*	*			
Ling, K. (2014)(26)	Commentary	Bilateral Regional	*	*			*
Lofters, A.K. (2012)(27)	Commentary	Bilateral Regional International	*	*	*		
Makulec, A (2014)(28)	Working Paper Series	Bilateral International	*	*	*		
Manning, C. (2007)(29)	Case study	Regional International	*				
Morgan, W.J. (2005)(30)	Case Study	International	*		*	*	
Nullis-Kapp, C. (2005)(31)	WHO News Bulletin	Bilateral International	*				
Ognyanova, D. (2012)(32)	Mixed-Methods Qualitative Study	Bilateral Regional International	*		*	*	*
Pagett, C. (2007) (33)	Review	Bilateral Regional International	*	*	*	*	
Pastor‐Bravo, M. (2019)(34)	Case Study	Bilateral	*			*	*
Plotnikova, E.V. (2012)(35)	Document analysis & Qualitative Interviews	Bilateral Regional, International	*	*	*	*	*
Plotnikova, E.V. (2012)(36)	Qualitative Content Analysis	Bilateral Regional, International	*	*		*	*
Reardon, C. (2014)(37)	Qualitative study	Bilateral International	*				
Salmon, M.E. (2007)(38)	Program Report	Bilateral Regional	*	*			
Simoens, S. (2005)(39)	Working Paper	Bilateral Regional International	*				
Siyam, A (2013)(40)	Progress report	Bilateral Regional International	*	*	*		*
Squires, A. (2011)(41)	Qualitative study	Regional	*	*			
Stilwell, B. (2004)(42)	Commentary	Bilateral Regional International	*			*	
Tangcharoensathien, V. (2015)(43)	Commentary	Bilateral Regional International	*	*			
Tangcharoensathien, V. (2017)(44)	Qualitative Policy analysis	Bilateral International	*		*		
Tankwanchi, A.B.S (2014)(45)	Correspondence	International	*				
Te, V. (2018)(46)	Scoping Review	Regional International	*	*	*	*	
Tsujita, Y. (2017)(47)	Research Report	Bilateral Regional	*			*	*
Villegas, B.M (1993)(48)	Narrative Review	Regional	*			*	
Walton-Roberts, M.J. (2020)(49)	Book Chapter	Bilateral, International	*				
Wangchuk, K. (2015)(50)	Brief Communication	Bilateral Regional	*				
Willetts, A. (2004)(51)	Review	International	*	*		*	
World Health Organization, Regional Office for The Western Pacific (2003)(52)	Meeting Report	Bilateral Regional, International	*		*		*
WHO (2003)(53)	Report	International	*				*
World Health Organization (2006)(54)	Report	Bilateral Regional International	*			*	
World Health Organization Regional Committee for Africa. (2009)(55)	Technical Report	International	*	*	*	*	*
World Health Organization Regional Office for South-East Asia. (2009)(56)	Technical Paper	International Bilateral	*	*		*	*
World Health Organization Regional Office for the Western Pacific. (2003)(57)	Meeting Report	Bilateral Regional, International	*		*		
World Health Organization Regional Office for the Western Pacific (2013)(58)	Analytical Review	Regional, International	*		*		
Yagi, N. (2014)(59)	Policy Analysis	Bilateral Regional International	*	*	*	*	
Yeates, N (2013)(60)	International Policy Report	Bilateral Regional International	*	*	*	*	
Yeates, N. (2018)(61)	Analytical Review	Bilateral Regional International	*		*	*	*
Yeates, N. (2019)(62)	Research Monograph	Bilateral Regional International	*	*	*	*	*
Young, R. (2013)(63)	Evaluation Study (Mixed Methods)	Bilateral International	*	*	*	*	

**Key**: PVP – public value proposition, AE – authorising environment, OPC – operational capacity, CPV – competing public values, HaCPV – handling of competing public values. ^¥ -^ Articles mentioned at least one CPV or an approach for HaCPV

**References**

1. Abuagla A, Badr E. Challenges to implementation of the WHO Global Code of Practice on International Recruitment of Health Personnel: the case of Sudan. Human Resources for Health. 2016;14(Supplement 1):26.

2. Arnold PC. Why the ex-colonial medical brain drain? Journal of the Royal Society of Medicine. 2011;104(9):351–4.

3. Balasubramanian M, Brennan DS, Spencer AJ, Short SD. The international migration of dentists: directions for research and policy. Community Dentistry and Oral Epidemiology. 2016;44(4):301–12.

4. Balasubramanian M, Short S. The Commonwealth as a custodian of dental migratory ethics: Views of senior oral health leaders from India and Australia. International Dental Journal. 2011;61(5):281–6.

5. Bidwell P, Laxmikanth P, Blacklock C, Hayward G, Willcox M, Peersman W Security and skills: the two key issues in health worker migration. Global Health Action. 2014;7:24,194.

6. Brush BL. Global nurse migration today. Journal of Nursing Scholarship. 2008;40(1):20–5.

7. Brush BL. The potent lever of toil: nursing development and exportation in the postcolonial Philippines. American Journal of Public Health. 2010;100(9):1572–81.

8. Buchan J. Challenges for WHO code on international recruitment. BMJ. 2010;340:c1486.

9. Buchan J. The financial cost of physician emigration from sub-Saharan Africa. BMJ (Online). 2011;343(7835):d6817.

10. Buchan J, Parkin T, Sochalski J. International nurse mobility: trends and policy implications. [cited 20 July 2021]. Available from: https://www.who.int/workforcealliance/knowledge/resources/nursesmobility/en/

11. Cheng MH. The Philippines’ health worker exodus. The Lancet. 2009;373(9658):111–2.

12. Connell J, Buchan J. The impossible dream? Codes of Practice and the international migration of skilled health workers. World Medical and Health Policy. 2011;3(3):3.

13. Cowan D, Wilson-Barnett DJ. Yes European Healthcare Training and Accreditation Network (EHTAN) project. International Journal of Nursing Studies. 2006;43(3):265–7.

14. Dhillon IS, Clark ME, Kapp RH. Innovations in cooperation: A Guidebook on bilateral agreements to adress health worker migration [cited 26 July 2021]. Available from: https://www.aspeninstitute.org/publications/innovations-cooperation-guidebook-bilateral-agreements-address-health-worker-migration/

15. Ennis CA, Walton-Roberts M. Labour market regulation as global social policy: The case of nursing labour markets in Oman. Global Social Policy. 2018;18(2):169–88.

16. World Health Organization Regional Office for Europe European Observatory on Health Systems and Policies. Health professional mobility in a changing Europe [cited 16 July 2021]. Available from: https://apps.who.int/iris/handle/10665/326372

17. Gerlinger T, Schmucker R. Transnational migration of health professionals in the European Union. Cadernos de Saude Publica. 2007;23(SUPPL.2):S184-S92.

18. Hawthorne L. International Health Workforce Mobility and Its Implications in the Western Pacific Region: World Health Organization; 2015 [cited 16 July 2021]. Available from: https://findanexpert.unimelb.edu.au/scholarlywork/1037803-international-health-workforce-mobility-and-its-implications-in-the-western-pacific-region

19. Jourdain A, Pham T. Mobility of physicians in Europe: health policies and health care provision. Sante publique (Vandoeuvre-les-Nancy, France). 2017;29(1):81–7.

20. Kalipeni E, Semu LL, Mbilizi MA. The brain drain of health care professionals from sub-Saharan Africa: a geographic perspective. Progress in Development Studies. 2012;12(2/3):153–171.

21. Kanchanachitra C, Lindelow M, Johnston T, Hanvoravongchai P, Lorenzo FM, Huong NL Human resources for health in southeast Asia: Shortages, distributional challenges, and international trade in health services. The Lancet. 2011;377(9767):769–81.

22. Kingma M. Nursing migration: global treasure hunt or disaster-in-the-making? Nursing inquiry. 2001;8(4):205–12.

23. Kingma M. New challenges, emerging trends, and issues in regulation of migrating nurses. Policy, politics & nursing practice. 2006;7(3 Suppl):26S-33S.

24. Kupfer L, Hofman K, Jarawan R, McDermott J, Bridbord K. Strategies to discourage brain drain. Bulletin of the World Health Organization. 2004;82(8):616–23.

25. Lewis P. Training nurses for export: A viable development strategy? Social and Economic Studies. 2011;60(2):67–104.

26. Ling K, Belcher P. Medical migration within Europe: Opportunities and challenges. Clinical Medicine Journal of the Royal College of Physicians of London. 2014;14(6):630–2.

27. Lofters AK. The ‘brain drain’ of health care workers: Causes, solutions and the example of Jamaica. Canadian Journal of Public Health. 2012;103(5):376–8.

28. Makulec A. Philippines’ Bilateral Labour Arrangements on Health-care Professional Migration: In Search of Meaning. [cited 19 July 2021]. Available from: http://www.ilo.org/manila/publications/WCMS_320609/lang–en/index.htm

29. Manning C, Sidorenko A. The Regulation of Professional Migration: Insights from the Health and IT Sectors in ASEAN. The World Economy. 2007;30(7):1084–113.

30. Morgan WJ, Sives A, Appleton S. Managing the International Recruitment of Health Workers and Teachers: Do the Commonwealth Agreements Provide an Answer? The Round Table. 2005;94(379):225–38.

31. Nullis-Kapp C. Efforts under way to stem ‘brain drain’ of doctors and nurses. Bulletin of the World Health Organization. 2005;83(2):84–5.

32. Ognyanova D, Maier CB, Wismar M, Girasek E, Busse R. Mobility of health professionals pre and post 2004 and 2007 EU enlargements: Evidence from the EU project PROMeTHEUS. Health Policy. 2012;108(2–3):122–32.

33. Pagett C, Padarath A. A review of codes and protocols for the migration of health workers. Harare: HST, EQUINET, ECSA HC: EQUINET; 2007 [cited 16 July 2021]. Available from: https://www.aspeninstitute.org/wp-content/uploads/files/content/images/review%20of%20codes%20and%20protocols%20pagett.pdf

34. Pastor‐Bravo M, Nelson S. Migration of Latin American nurses to Spain 2006–2016: a case study. International Nursing Review. 2019;66(2):183–90.

35. Plotnikova E. Recruiting foreign nurses for the UK: the role of bilateral labour agreements2012 [cited 16 July 2021]. Available from https://era.ed.ac.uk/handle/1842/6445?show=full

36. Plotnikova EV. Cross-border mobility of health professionals: Contesting patients’ right to health. Social Science and Medicine. 2012;74(1):20–7.

37. Reardon C, George G. An examination of the factors fueling migration amongst Community Service practitioners. African Journal of Primary Health Care and Family Medicine. 2014;6(1).

38. Salmon ME, Yan J, Hewitt H, Guisinger V. Managed migration: The Caribbean approach to addressing nursing services capacity. Health Services Research. 2007;42(3):1354–72.

39. Simoens S, Villeneuve M, Hurst J. Tackling nurse shortages in OECD countries2005 [cited 16 November 2021]. Available from https://pdfs.semanticscholar.org/89a2/832a05651ee0e5116ed2fc4571ed066b88af.pdf?_ga=2.82453598.910677897.1600083369–1680489653.1599476236

40. Siyam A, Zurn P, RÃ¸ OC, Gedik G, Ronquillo K, Co CJ Monitoring the implementation of the WHO Global Code of Practice on the International Recruitment of Health Personnel. Bulletin of the World Health Organization. 2013;91(11):816–23.

41. Squires A. The North American Free Trade Agreement (NAFTA) and Mexican Nursing. Health Policy and Planning. 2011;26(2):124–32.

42. Stilwell B, Diallo K, Zurn P, Vujicic M, Adams O, Dal Poz MR. Migration of health-care workers from developing countries: strategic approaches to its management. Bulletin of the World Health Organization 2004;82(8):595–600.

43. Tangcharoensathien V, Travis P. Accelerate Implementation of the WHO Global Code of Practice on International Recruitment of Health Personnel: Experiences From the South East Asia Region: Comment on ‘Relevance and Effectiveness of the WHO Global Code Practice on the International Recruitment of Health Personnel - Ethical and Systems Perspectives’. Int J Health Policy Manag. 2015;5(1):43–6.

44. Tangcharoensathien V, Travis P, Tancarino AS, Sawaengdee K, Chhoedon Y, Hassan S Managing In- and Out-Migration of Health Workforce in Selected Countries in South East Asia Region. International Journal of Health Policy and Management. 2017;7(2):137–43.

45. Tankwanchi ABS, Vermund SH, Perkins DD. Has the WHO Global Code of Practice on the International Recruitment of Health Personnel been effective? The Lancet Global Health. 2014;2(7):e390-e1.

46. Te V, Griffiths R, Law K, Hill PS, Annear PL. The impact of ASEAN economic integration on health worker mobility: a scoping review of the literature. Health Policy and Planning. 2018;33(8):957–65.

47. Tsujita Y. Human Resource Development and the Mobility of Skilled Labour in Southeast Asia: The Case for Nurses 2017 [cited 16 July 2021]. Available from: https://www.ide.go.jp/English/Publish/Reports/Brc/19.html

48. Villegas BM. Implications of AFTA on Philippine labor export. Asian and Pacific Migration Journal 1993;2(3):285–301.

49. Walton-Roberts M. The Production of Nurses for Global Markets: Tracing Capital and Labour Circulation In and Out of Asia. Cambridge: Cambridge University Press; 2020 [cited 16 July 2021]. Available from: https://www.cambridge.org/core/books/mobilities-of-labour-and-capital-in-asia/production-of-nurses-for-global-markets-tracing-capital-and-labour-circulation-in-and-out-of-asia/ECE5CAAC7DB071F9D6DF398260AAD1BE

50. Wangchuk K, Supanatsetakul N. Foreign medical practitioners: requirements for medical practice and postgraduate training in Thailand under ASEAN Economic Community liberalization in 2015. Asian Biomedicine. 2015;9(6):777–782.

51. Willetts A, Martineau T. Ethical international recruitment of health professionals: Will codes of practice protect developing country health systems? [cited 16 November 2021]. Available from: https://www.aspeninstitute.org/wp-content/uploads/files/content/images/Martineau%20codesofpracticereport.pdf

52. Buchan J, Parkin T, Sochalski J. International nurse mobility: trends and policy implications. [cited 20 July 2021]. Available from: https://www.who.int/workforcealliance/knowledge/resources/nursesmobility/en/

53. World Health Organization. International migration, health and human rights 2003 [cited 16 July 2021]. Available from: https://apps.who.int/iris/bitstream/handle/10665/42793/9241562536.pdf?sequence = 1

54. World Health Organization. Treat, train, retain: the AIDS and health workforce plan: report on the Consultation on AIDS and Human Resources for Health, WHO, Geneva, 11–12 May 2006 [cited 26 November 2021]. Available from: https://apps.who.int/iris/handle/10665/43558

55. World Health Organization Regional Committee for Africa. International Recruitment of Health Personnel - Draft Global Code of Practice 2009 [cited 17 July 2021]. Available from: https://apps.who.int/iris/handle/10665/1800?locale-attribute=ru&

56. World Health Organization Regional Office for South-East Asia. Code of practice for the international recruitment of health personnel: New Delhi: WHO Regional Office for South-East Asia.; 2009 [cited 16 July 2021]. Available from: https://apps.who.int/iris/handle/10665/127739

57. World Health Organization Regional Office for the Western Pacific. Meeting on Migration of Skilled Health Personnel in Pacific Island Countries, Nadi, Fiji, 23–27 June 2003. Manila: WHO Regional Office for the Western Pacific; 2003 [cited 17 July 2021]. Available from: https://apps.who.int/iris/handle/10665/208672

58. World Health Organization Regional Office for the Western Pacific. Implementation of the human resources for health strategy in the Western Pacific Region: An analytical review. [cited 17 July 2021]. Available from: https://apps.who.int/iris/handle/10665/208194

59. Yagi N, Mackey TK, Liang BA, Gerlt L. Policy review: Japan-Philippines Economic Partnership Agreement (JPEPA)-analysis of a failed nurse migration policy. International Journal of Nursing Studies. 2014;51(2):243–50.

60. Yeates N, Pillinger J. Human Resources for Health Migration: global policy responses, initiatives, and emerging issues.2013 [cited 23 July 2021]. Available from: http://oro.open.ac.uk/39072/

61. Yeates N, Pillinger J. International healthcare worker migration in Asia Pacific: International policy responses. Asia Pacific Viewpoint. 2018;59(1):92–106.

62. Yeates N, Pillinger J. International Health Worker Migration and Recruitment: Global Governance, Politics and Policy. London: Routledge; 2019 [cited 16 July 2021]. Available from: https://doi.org/10.4324/9781315678641

63. Young R. How effective is an ethical international recruitment policy? Reflections on a decade of experience in England. Health Policy. 2013;111(2):184–92.

## Appendix 3 Code book for Scoping Review: Governance Systems for SHW migration & Public Value

**Table:**

Item and its interpretation	Code
1. Level of migration governance:	
• Bilateral agreements/initiatives: If the governance decisions (for skilled health migration) and/or their implementation was between two countries only.	Bil.Agree/Init
• Regional agreements/initiatives: If the governance decisions (for skilled health migration) and/or their implementation was within a region (i.e. between countries that have shared characteristics e.g. Africa, Europe, Asia, Europe, North America etc)	Reg.Agree/Init
• International agreements/initiatives: If the governance decisions (for skilled health migration) and/or their implementation was between three or more countries that do not necessarily share the same characteristics (e.g. between countries in Africa, and those in Europe)	Int.Agree/Init
2. What was the public value proposition offered by agreement/governance initiative? Look for the main vision or desired outcome of the agreement. Also look for the mission (i.e. steps taken to achieve the overall vision), strategic goals that were stated for each skilled health workforce migration policy or initiative	Main.Aim
3. Were there competing public values (i.e. competing aims between participating countries)? If yes, please code them. 4. Examples of public values are as follows: economic values – speaks to economic gains for the country/organization and the citizens, social and cultural value – speaks to social capital/cohesion, what binds people together, breeds trust and commitment to one another, political value – what increases democratic dialogues and public participation, ecological value – what preserves the environment, reduces waste and ensures sustainable development, social value – from the perspective of citizens as they use public health services. Protecting citizen’s/migrant rights is another public value.	Economic values: Pub.Val_Econ Social & cultural value:Pub.Val_Soc.Cult Political value:Pub.Val_Pol Ecological value:Pub.Val_Ecol Social value:Pub.Val_Soc Protecting citizen’s/migrant rights: Pub.Val_Rights
5. How were these competing public values handled? Codes for this section include: • Cooperation (Solidarity)- look for evidence on whether participating countries/organizations tried to forge a shared sense of identity, partnership and collective action towards a common interest. • Autonomy – look for evidence of competition between countries for scarce skilled health workers, where singular national/organizational interests was the focus instead of the collective, whether there was so much focus on self-determination or sovereignty of participating countries/organizations. • Equality and equity – look for evidence for the utilization of standards for guiding decision making, including ethical standards, codes of conduct, consultation between participating countries to ensure transparency and accountability. 6. Authority – look for evidence for the presence of an arrangement for enforcing regulatory mechanisms; their reliability and efficiency.	Solidarity: Comp.Pub.Val_Sol Autonomy:Comp.Pub.Val_Aut Equality and Equity:Comp.Pub.Val_EE Authority:Comp.Pub.Val_Auth
7. Enabling and Constraining Factors? Look for information on what enabled or constrained the attainment of the public value propositions and the ability to address competing priorities.	Ena/Cons
8. Provide information on what changed concerning health professional migration between participating countries. What changed concerning the migration of health professionals between these countries?	Chang.Mig

**Preferred Reporting Items for Systematic reviews and Meta-Analyses extension for Scoping Reviews (PRISMA-ScR) Checklist**

**Table:**

SECTION	ITEM	PRISMA-ScR CHECKLIST ITEM	REPORTED ON PAGE #
TITLE			
Title	1	Identify the report as a scoping review.	1
ABSTRACT			
Structured summary	2	Provide a structured summary that includes (as applicable): background, objectives, eligibility criteria, sources of evidence, charting methods, results, and conclusions that relate to the review questions and objectives.	2
INTRODUCTION			
Rationale	3	Describe the rationale for the review in the context of what is already known. Explain why the review questions/objectives lend themselves to a scoping review approach.	4
Objectives	4	Provide an explicit statement of the questions and objectives being addressed with reference to their key elements (e.g. population or participants, concepts, and context) or other relevant key elements used to conceptualize the review questions and/or objectives.	5
METHODS			
Protocol and registration	5	Indicate whether a review protocol exists; state if and where it can be accessed (e.g. a Web address); and if available, provide registration information, including the registration number.	5
Eligibility criteria	6	Specify characteristics of the sources of evidence used as eligibility criteria (e.g. years considered, language, and publication status), and provide a rationale.	6
Information sources*	7	Describe all information sources in the search (e.g. databases with dates of coverage and contact with authors to identify additional sources), as well as the date the most recent search was executed.	7
Search	8	Present the full electronic search strategy for at least 1 database, including any limits used, such that it could be repeated.	Appendix 1
Selection of sources of evidence†	9	State the process for selecting sources of evidence (i.e. screening and eligibility) included in the scoping review.	7
Data charting process‡	10	Describe the methods of charting data from the included sources of evidence (e.g. calibrated forms or forms that have been tested by the team before their use, and whether data charting was done independently or in duplicate) and any processes for obtaining and confirming data from investigators.	8
Data items	11	List and define all variables for which data were sought and any assumptions and simplifications made.	8
Critical appraisal of individual sources of evidence§	12	If done, provide a rationale for conducting a critical appraisal of included sources of evidence; describe the methods used and how this information was used in any data synthesis (if appropriate).	N/A
Synthesis of results	13	Describe the methods of handling and summarizing the data that were charted.	8
RESULTS			
Selection of sources of evidence	14	Give numbers of sources of evidence screened, assessed for eligibility, and included in the review, with reasons for exclusions at each stage, ideally using a flow diagram.	Figure 1
Characteristics of sources of evidence	15	For each source of evidence, present characteristics for which data were charted and provide the citations.	Appendix 2
Critical appraisal within sources of evidence	16	If done, present data on critical appraisal of included sources of evidence (see item 12).	N/A
Results of individual sources of evidence	17	For each included source of evidence, present the relevant data that were charted that relate to the review questions and objectives.	Appendix 2
Synthesis of results	18	Summarize and/or present the charting results as they relate to the review questions and objectives.	Table 1, Figures 3–5
DISCUSSION			
Summary of evidence	19	Summarize the main results (including an overview of concepts, themes, and types of evidence available), link to the review questions and objectives, and consider the relevance to key groups.	17
Limitations	20	Discuss the limitations of the scoping review process.	21
Conclusions	21	Provide a general interpretation of the results with respect to the review questions and objectives, as well as potential implications and/or next steps.	21
FUNDING			
Funding	22	Describe sources of funding for the included sources of evidence, as well as sources of funding for the scoping review. Describe the role of the funders of the scoping review.	23

JBI = Joanna Briggs Institute; PRISMA-ScR = Preferred Reporting Items for Systematic reviews and Meta-Analyses extension for Scoping Reviews. * Where *sources of evidence* (see second footnote) are compiled from, such as bibliographic databases, social media platforms, and Web sites. † A more inclusive/heterogeneous term used to account for the different types of evidence or data sources (e.g. quantitative and/or qualitative research, expert opinion, and policy documents) that may be eligible in a scoping review as opposed to only studies. This is not to be confused with *information sources* (see first footnote). ‡ The frameworks by Arksey and O’Malley (6) and Levac and colleagues (7) and the JBI guidance (4, 5) refer to the process of data extraction in a scoping review as data charting. § The process of systematically examining research evidence to assess its validity, results, and relevance before using it to inform a decision. This term is used for items 12 and 19 instead of ‘risk of bias’ (which is more applicable to systematic reviews of interventions) to include and acknowledge the various sources of evidence that may be used in a scoping review (e.g. quantitative and/or qualitative research, expert opinion, and policy document). *From*: Tricco AC, Lillie E, Zarin W, O’Brien KK, Colquhoun H, Levac D PRISMA Extension for Scoping Reviews (PRISMAScR): Checklist and Explanation. Ann Intern Med. 2018;169:467–473. doi: 10.7326/M18-0850.
